# Long-term Clinical Results of Vitrectomy and Scleral Buckling in Treatment of Rhegmatogenous Retinal Detachment

**DOI:** 10.1155/2019/5416806

**Published:** 2019-03-06

**Authors:** I. Schmidt, N. Plange, G. Rößler, H. Schellhase, A. Koutsonas, P. Walter, B. Mazinani

**Affiliations:** ^1^Department of Ophthalmology, RWTH Aachen University, Aachen, Germany; ^2^Department of Ophthalmology, St. Martinus-Hospital, Düsseldorf, Germany

## Abstract

**Purpose:**

Most studies about retinal detachment cover a limited follow-up period. The purpose of this research is to assess the long-term results after pars plana vitrectomy (PPV) and scleral buckle (SB) surgery in patients with rhegmatogenous retinal detachment (RRD).

**Methods:**

155 patients with RRD are treated either with SB or PPV with a mean follow-up of more than 5 years. Retrospective analysis of patient data with RRD was performed between January 2006 and June 2008 at a tertiary eye clinic.

**Results:**

Overall primary success rate was 85.2% (PPV: 84.6%, SB: 89.5%; p=0.57). 90.5% of redetachments appeared within the first 124 days. No significant different success rate was found for vitrectomy with and without additional encircling band (p=0.09). No advantage of a supplemental encircling band in cases of preoperative inferior breaks was seen (p=0.81). Patients of SB group were treated more frequently in follow-up time because of epiretinal membrane (ERM) (SB: 15.5% versus PPV: 7.3%). No patient of the PPV group without intraoperative use of endolaser cerclage (14.7%) had any peeling surgery postoperatively.

**Conclusion:**

Redetachment rates of both methods are comparable in a clinical setting where PPV is considered a suitable method for pseudophakic patients and in complex cases and SB was performed in younger phakic patients with clearly identified retinal tears. PPV seems to show a more heterogenous pattern of complications. No advantage of a supplemental encircling band could be found in these case series of patients with primary RRD. No relevant long-term risk of redetachment was seen after SB.

## 1. Introduction

Scleral buckling (SB) and pars plana vitrectomy (PPV) have shown similar functional and anatomical results in the treatment of retinal detachment. However, most of the studies only cover a short follow-up period [1–6]. This retrospective clinical follow-up study is set up to show short- and long-term results and clinical outcome of retinal detachment surgery in a regular setting of a tertiary eye clinic. Both surgical methods should be checked regularly for their applicability and then put into context with the real situation. This study was prepared in order to record and analyze the long-term course after a retinal detachment surgery at a time when the vitrectomy seems to displace the hump technique more and more. Secondly this study is intended to answer if eyes, treated with scleral buckle only, develop more frequently a long-term recurrence of retinal detachment because of the remaining of the pathologic vitreous body with its potential tractional forces. Furthermore, this study was designed to investigate if there is a benefit of vitrectomy with an additional encircling band in cases with inferior located retinal breaks. This study was performed to analyze real-life data in the surgical management of retinal detachment. As the indications of the surgical procedures are in part different as described in the methods sections, no randomization or direct comparison of both methods is warranted.

## 2. Material and Methods

This retrospective analysis is performed to inform about the long-term results of scleral buckling and pars plana vitrectomy in patients with a primary rhegmatogenous retinal detachment (RRD) up to medium complexity. Before the data analysis took place, ethics committee approval in accordance with the Declaration of Helsinki was obtained. Data from patients who were treated because of primary RRD between January 2006 and June 2008 were collected retrospectively and analyzed after application of exclusion criteria. Patients with uncomplicated RRD were enrolled in the study. Patients with a proliferative vitreoretinopathy (PVR) grade B or C, giant tears, macular holes, or previous retinal surgery were excluded [1]. Tractional-, serous-, and posttraumatic detachments were also ruled out of the analysis. To obtain a more homogenous patient collective, complicated retinal conditions that primarily required an oil-endotamponade were also factored out. Finally, the study included 155 patients with a mean follow-up period of more than 5 years. For the data collection, the information of patients file was used. Pre-, intra-, and postoperative findings were documented. Based on fundus drawings and notes of surgical reports, detailed information was collected concerning pre- and intraoperative findings. To get informed about long-term clinical outcome after the in-patient treatment, the resident ophthalmologists were asked to complete a questionnaire. With the help of this questionnaire, we received information on the following questions:

- What was the last BCVA of the patient?
- Were further eye operations performed after the last presentation in our clinic?
- Was the retina attached or detached at the last visit, was the retinal attachment under oil?
- Were there any complications?

The choice, of which technique to use for surgical retinal detachment repair, was based on multiple criteria and was taken by experienced ophthalmic surgeons in an individualized manner. In general, patients with a large retinal hole and/or multiple retinal holes and advanced retinal detachment, especially when these were located on the posterior pole, underwent a vitrectomy. In addition, pseudophakic patients, who are known to often have small, anteriorly located holes initially undetected were treated using PPV. In case of an unknown initial hole situation the decision was always taken to use a vitrectomy. If the retinal detachment was spread out over more than one quadrant, the decision was also taken to use PPV. If preoperatively, there was a beginning PVR (grade A), higher PVR were not included in the study, and the patients underwent vitrectomy. In summary, reasons for a vitrectomy were

1. Pseudophakic status
2. PVR
3. Large retinal holes, multiple holes, and unclear hole situation
4. Detached Macula

The decision of the additional use of an encircling band or an endolaser cerclage was also performed on an individual basis, with special focus on more complicated situations with inferior breaks or intraoperative adherent vitreous body. However, some surgeons used the methods routinely. An additional encircling band was used in 64% patients of the PPV group and in 85,3% an endolaser cerclage was applied.

In contrast, a scleral buckle was performed in younger phakic patients with clearly visible retinal holes that were located in a manner that could be managed with a single silicone sponge.

## 3. Parameters

General clinical data like age, gender, duration of symptoms, lens status (phakic, pseudophakic), presence of high myopia (i.e., myopia <-6 dpt), and BCVA converted into Logarithm of the Minimum Angle of Resolution (LogMAR); preoperatively and at last follow-up was collected. The preoperative retinal situation was documented in detail (extension of the detachment, number and location of retinal breaks, presence of retinal breaks between the 4 and 8 o'clock position (i.e., inferior breaks), involvement of the macula, and proliferative vitreoretinopathy). Due to a lack of data, no statistical analysis concerning myopia could be performed. With the help of the surgical reports, the respective surgery method (scleral buckle or pars plana vitrectomy with SF6 20% (sulfur hexafluorid) gas endotamponade) was reported. If in the vitrectomy group an additional encircling band was used, this was also considered and further subdivided into a patient group with inferiorly located holes. The use of intraoperative endolaser cerclage, cryotherapy or drainage of subretinal fluid and/or intraoperative gas endotamponade during scleral buckling, the surgeon and eventual complications were listed. Retinal attachment after single surgery till the last follow up visit was defined as primary anatomical success. Long-term findings like BCVA, phthisis or enucleation as most serious long-term complication after surgery, meanwhile performed surgeries, and retinal situation (retina attached, persisting retinal detachment, and retinal attachment under oil endotamponade) were documented and analyzed. The latency of recurrence of a retinal detachment was analyzed. Only clinically relevant epiretinal membrane (ERM) or cataract development requiring surgery was reported. Occurrence of a potential retinal detachment of the fellow eye was analyzed as well. Follow-up time and long-term data were noticed with the help of patients file and the data of questionnaires filled out by the resident ophthalmologists, in 77.4% completely filled out questionnaires, existed.

## 4. Surgical Procedure

In scleral buckle surgery, the conjunctiva was opened circularly. Then the four recti muscles were threaded. Using indirect ophthalmoscopy, the retinal tear was located and treated with cryotherapy. After that an encircling band or a single silicone sponge was sewed on the sclera to dent the bulb for reaching retinal attachment. Drainage of subretinal fluid or intravitreal air injection was performed if necessary.

Circular conjunctival opening was also the first step in surgery of the pars plana vitrectomy group. All patients included in this study were treated using 20gauge pars plana vitrectomy. Scleral incisions were made in a distance of 3.0-3.5mm from the corneoscleral limbus. Next step was placing the infusion port, light, and surgical instruments. Afterwards a complete vitrectomy was performed. Retinal attachment could be achieved with the help of perfluorocarbon liquid (PFCL). After identification of all retinal breaks, endolaser and/or cryotherapy and endolaser cerclage were used to create a retinal scar. Endolaser cerclage was performed with initial power settings of 100 milliseconds and 100 milliwatt, individually adjusted to achieve three rows of light grey lasers pots on the retina. Then, PFCL-air exchange took place and sclerotomies were closed. Last step was filling the eye with SF6 20% gas endotamponade and removing the infusion.

## 5. Statistical Analysis

The study data were documented in Excel tables and analyzed using the SPSS program (Statistical Package for Social Sciences for Windows v.22.0, SPSS Inc. Chicago, IL). Nominal scaled data were presented as percentages and statistical significance was tested with the help of cross tables and Pearson's chi-squared.test. For data scaled metrically the One-Way analysis of variance (One-Way ANOVA) was used. The level of statistical significance was considered as p<0,05.

## 6. Results

One hundred and fifty-five eyes were included (male: 90 patients, 58.1%/female: 65 patients, 41.9%). The mean age was 62.1 ±13.1 years, and the scleral buckle group was on average 4.5 years younger than the vitrectomy group. The mean follow-up time was 5.45±2.55 years, median was 6.17 years, the shortest follow-up period was 6 months, and the longest was 9.46 years. The mean follow-up time of the scleral buckle group was 67.4 months (5.6 years) ±35 months, median was 77.8 months (6.5 years), the vitrectomy group had a mean follow-up time of 64.9 ± 30.3 months (5.4 years), and median was 73.5 months. The follow-up time did not differ significantly (p=0.74). 93 patients (60%) were phakic. Pseudophakia was significantly higher represented in the vitrectomy group (n= 61 in PPV group versus n= 1 in SB group, p=0.001). The majority of patients presented after 14.8±37.7 days of symptom duration.

There was a statistically significant difference (p=0.007) of preoperative BCVA between the groups. BCVA of scleral buckle patients was on average 0.5 logMAR before surgery, compared to vitrectomy patients with a BCVA of 1.0 logMAR. Retinal detachment had an extension of 1 quadrant in 30 eyes (19.4%), 85 eyes (54.8%) presented with a detachment of 2 quadrants, 26 eyes (16.8%) had an extension of 3 quadrants, and a total retinal detachment was seen in 14 eyes (9.0%). Patients in the SB group had a maximum extension of 2 quadrants. The presence of a preoperative macular detachment was significantly higher in the vitrectomy group (SB: n=2 versus PPV: n=59, p<0.0001). Overall, 94 (60.6%) presented with an attached macula. In the scleral buckle group 1.3 retinal breaks were detected on average, whereas a mean of 2.4 retinal breaks was found in the vitrectomy group (p=0.002). The maximum number of retinal breaks in the vitrectomy group was 8 versus 4 retinal breaks in the scleral buckle group. 55 of 155 patients (35.5%) had inferior breaks. Preoperative PVR was analyzed with the help of fundus drawings. 26 (19%) patients of the vitrectomy group had a PVR reaction preoperatively; in comparison, in the scleral buckle group only one patient (5.1%) presented with a PVR reaction (p=0.14).

Overall, 19 patients were treated with scleral buckle, 71% of these were treated using an encircling band and 29% with a single sponge only. Intraoperative catalysis puncture to drain subretinal fluid was performed in 10.5% of patients, and 5 patients (26.3%) had an additional gas injection. Vitrectomy was applied in 136 eyes, and around half of them (77 patients, 56.6%) were treated with an additional encircling band. Most patients received (85.3%) supplemental endolaser cerclage. Intra- and/or postoperative complications within the primary surgery occurred in 26.5% (Table 1).

The primary success rate was defined as a retinal reattachment after single surgery. This was achieved in 132 patients (85.2%). Respectively, vitrectomy had a success rate of 84.6% and scleral buckle a rate of 89.5% (p=0.57). Patients receiving vitrectomy with additional encircling band showed a higher primary anatomical success rate compared to vitrectomy alone, not reaching statistical significance (p=0.09). Regarding the vitrectomized patients with preoperative inferior breaks of the retina, primary anatomical success did not differ using a supplemental encircling band or not (p=0.81). No relevance in success rate was found in vitrectomized patients with supplemental endolaser cerclage. (p=0.95).

The median latency till onset of a recurrence of retinal detachment was 24 days. Mean was 227 ± 587 days (range 6-2463 days).

Regarding the scleral buckle group, 10.5% (2 patients) showed a recurrence of retinal detachment: one appeared after 483 days (15.9 months) and the other after 23 days (0.8 months, mean: 253 days). The vast majority of retinal redetachments (90.5%, 19 patients) of the vitrectomy group appeared during the first 124 days (4.1 months). The shortest latency of second retinal detachment in vitrectomy group was 6 days (0.2 months), and the longest was 2463 days (81 months). Long-term retinal attachment, defined as an attached retina at the last documented follow-up visit, was achieved in 149 patients (96.1%). 3.2% of the eyes had a silicone oil endotamponade at long-term follow-up examination to achieve retinal attachment. No enucleation or phtisis was noticed as most serious complication. Retinal attachment after a single surgery could be found in 89.5% of patients in the scleral buckle group. 5.3% needed two retinal surgeries and 5.3% had four surgeries to achieve anatomical success. 84.6% of the vitrectomy group had a single surgery leading to an attached retina. 4.4% of the patients had two surgeries, 8.1% three, 2.2% four and 1.36% five. In the last follow-up examination, SB group patients achieved a significantly better visual acuity with 0.2 logMAR compared to the vitrectomy group with 0.5 logMAR (p=0.031). The respective change of visual acuity showed no statistical difference (SB: -0.2 logMAR versus PPV: -0.44 logMAR, p=0.29). Another aspect analyzed in this study was the occurrence of ERM after using endolasercerclage in the primary surgery. In 85.3% of the overall cases, endolasercerclage was used. 8.6% of the patients developed a vision affecting ERM subsequently. Patients without an intraoperative use of endolasercerclage (14.7%) had no form of ERM treated with macular surgery. The postoperative development of a symptomatic cataract could be analyzed related to subsequently performed cataract surgery. 52 of 93 (56%) initially phakic patients had a cataract surgery after a certain time following the retinal surgery. Of these, 44 patients were (58.7%) in the vitrectomy group and 8 patients (44.4%) in the scleral buckle group. Time latency till cataract surgery in the respective groups was analyzed to compare the progress of cataract development and differed significantly between the groups (PPV group:15.4 months versus SB group: 33.5 months; p=0.013). 15.8% (3 patients) of the scleral buckle group and 7.3% (10 patients) of the vitrectomy group had a membrane peeling surgery because of ERM. The respective latency time till membrane peeling did not differ significantly between the groups (PPV group: 15.5 months, SB group: 18 months). Retinal detachment in the first eye represents a risk factor for retinal detachment in the second eye. This study showed a prevalence of 7.1% for retinal detachment of the fellow eye during long-term follow-up. 3.5% occurred within one year, 7.1% in six years.

## 7. Discussion

Most studies only had a follow-up period of 6 months [2, 6] or 12 months [1, 4, 5]. To our knowledge, compared to other studies on RD surgery, this study's follow-up time is much longer with its average five-year period.

SB and PPV group did not differ significantly regarding success rate of surgical treatment of rhegmatogenous retinal detachment in this long-term follow-up study of more than 5 years. Primary reattachment was seen in 85.2% of the overall group, final reattachment was achieved in 96.1% of all patients. Another study from Germany which analyzed results of retinal detachment surgery 10 years earlier, namely from 1990-1997, showed a slightly lower primary attachment rate of 71.2% than our study [7].The improvement in surgical results can be accorded to the continuous technical developments that the pars plana vitrectomy method underwent due to its relatively recent invention in the 1970s compared to the older hump technique.

A study of Ahmadieh et al. with 6 months follow up showed a reattachment rate of 68.2 % after primary surgery in SB group and 62.6% in PPV group (only phakic patients were included). Final success rate was 85% in SB and 92% in PPV group. Main reason for redetachment was proliferative vitreoretinopathy. [2] A not randomized, multicenter study of Adelman et al. collected data with the help of questionnaires from 4179 patients with uncomplicated retinal detachments of 48 clinics of different countries. Success rate in phakic patients was higher in SB group than in patients who had vitrectomy (p=0.028). Pseudophakic patients had a lower final failure rate in PPV group. [1] Significant difference in success rate was found in a prospective, randomized study of Heimann et al. with a one year follow up period. Pseudophakic patients treated with SB had a worse reattachment rate than vitrectomized patients (53.4% vs. 72%). [2] Another prospective, randomized study showed a primary success rate of 83% in SB group versus 94% in PPV group (p=0.037), no significant difference could be found any longer in final reattachment rate. [8] Results of this study are comparable with data of literature, with a tendency to higher success rates. For a comparative assessment, the different indications for the respective surgical methods must be taken into consideration. In general, phakic patients with a clearly locatable break situation have been treated with scleral buckle in our setting. Complex situations, already in an advanced stage, or pseudophakic patients, are mostly treated with vitrectomy. Interestingly, 88% of all patients with RRD were treated with PPV in this clinical setting of consecutive patients. The thesis, that in scleral buckle group the remaining vitreous body with its tractional forces may be related to a higher risk of a recurrence of retinal detachment in the long-term follow-up could not be verified in this study.

Ahmadieh et al. found that most redetachments occurred after 2 months (84.5%). [9] Because of limited data in the scleral buckle group of this study, the evidence of the redetachment results is low. The dates of the two re-detachments are far apart. However, redetachment rate is very low in the SB group even though a long-term risk might be speculated due to the remaining vitreous body. Redetachment occurred on average after 24 days (0.8 months). In the vitrectomy group, over 90% of redetachments took place during first six months postoperatively though, only a very small share of patients developed redetachment after several years.

PPV was used in 88% of patients, only 12% were treated with SB. Patients of SB group had a retinal detachment of up to 2 quadrants preoperatively, more extended detachments were only treated with PPV. The highest reattachment rate (93.2%) could be found in patients with the smallest detachment extension (≤1 quadrant). Extended detachments (>3 quadrants) had a success rate of 57.1%. Similar results can be found in a work of Salicone et al. A significant, negative prognostic factor was preoperative macular detachment. [3] It can be assumed, that the probability of a detached macula and consequently a poor postoperative result rises with extension of retinal detachment. Macula detachment in the group with the largest retinal detachment was 85.7%.

In addition, this study included all surgeons, who performed detachment surgery during 2006-2008 in the RWTH Aachen University hospital (n=6). However, overall success rates were satisfactory, although surgeries performed during night shifts or surgeons with lower levels of experience were not excluded to represent a typical setting of a tertiary eye clinic. The question of an optimal setting for RD surgery is raised again, i.e. to perform RD surgery as soon as possible in a situation of impending macular detachment or to wait for a high-experienced retina specialist. Koch found in 2012, that a detachment surgery in emergency setting did not improve the anatomical outcome and that it even worsened if performed by non-expert-surgeons [10].

More than 70% of vitrectomy group patients had a visual acuity of >0,4 LogMAR at initial presentation. Most patients even presented with visual acuity >1 LogMAR. Whereas, only 30% of SB group patients had a visual acuity >0,4 LogMAR. Visual acuity is linked to the severity of retinal detachment. Macular detachment usually correlates with a significant decrease of vision. 43% of vitrectomized patients initially presented with detached macula, only in 10% of buckle patients the macula was detached. Additional risk factors for a poor visual outcome are total retinal detachment and presence of preoperative PVR. [11–15] Despite of different baseline conditions, postoperative visual results of both groups showed similar increase of visual acuity postoperatively. Heimann et al. showed in 2007, that phakic patients of SB group have a significantly greater visual acuity change in comparison to phakic vitrectomized patients. No difference was found in pseudophakic patients. [2]

Pseudophakia is a known risk factor for retinal detachment. 40 % of patients in this study were pseudophakic preoperatively. According to Olsen and Jeppesen, cataract surgery correlates with a 2.3 times higher risk for retinal detachment. Especially young aged, male patients with a high axis length and intraoperative complications are highly affected. [16] Characteristically, retinal breaks in pseudophakic retinal detachments are small, anterior located retinal breaks, which were first seen intraoperatively. [17] Vitrectomy should be the preferred method treating pseudophakic detachments due to the limited visibility of all retinal breaks preoperatively. According to Christensen and Villumsen, there is no difference in anatomical and functional outcome between phakic and pseudophakic detachments. [18] Similar results were seen in this study.

A secondary question in this study is the advantage of an additional encircling band in vitrectomized eyes, especially in subjects with retinal breaks of the inferior hemisphere. An additional encircling band in vitrectomy is meant to support the vitreous base and allow a better visualization of the periphery. The disadvantage of an additional encircling band is a higher invasivity of the surgical procedure, a longer duration of surgery and certain postoperative complications, especially in comparison to small gauge vitrectomy. A multicenter randomized study of Walter et al. with 257 patients and a 6 months follow-up showed that an additional encircling band does not reduce the risk of a recurrence of retinal detachment significantly. [19] In our study even patients with inferior located breaks did not profit from an additional scleral buckling.

The list of complications, in particular intraoperative complications, is far longer in the vitrectomy group compared to SB. Common complications are iatrogenic breaks during 20-gauge surgery. One of the main postoperative complications of the vitrectomy group was postsurgical macular edema (4.4%), Berrod et al. described a similar incidence. [12] The incidence of metamorphopsia in the SB and PVV group were respectively 5.2% and 5.1%. Compared to the literature the number of postoperative macular edema seems very low, Okamoto et al. proclaimed 22-33%, and another study stated even more with 67% [20, 21]. This might be due to the non-prospective evaluation in our study. A study of Lina et al. pointed out a similar macular recovering after surgery of both surgery techniques. A preoperative detached macula presents a risk factor for a development of metamorphopsia after surgery. [22] Another known complication of vitrectomy is the postoperative progression of cataract development. The SPR study showed a cataract development in 33% of patients treated RD with SB and in 53% of vitrectomized patients. [2] Oshima et al. also described cataract development in 53% after vitrectomy. [4] This study presents similar results with 58.7% cataract progression in vitrectomized patients, Cataract surgery was performed after a mean of 15 months. In the SB group, 44.4% had a cataract surgery after 33 months.

According to the literature, the incidence of a postoperative development of an ERM is 4-8% [9, 11, 23]. The incidence in the present study was 7-15%. Known risk factors for ERM are old age, preoperative vitreous hemorrhage, large retinal breaks, macular detachment, previous operations and intraoperative use of cryotherapy. [5, 24–27] Patients of SB group in this study were operated because of ERM more frequently (SB: 15.5% versus PPV: 7.3%), which may be explained by the intraoperative standard use of cryotherapy in SB surgery or the absence of the vitreous in the post vitrectomy group. No peeling surgery has been performed in the patients of the PPV group that did not receive endolasercerclage during surgery. In contrast, 11.6% of patients with endolasercerclage developed a significant gliosis. It is assumed that the intraoperative use of endolaser leads to an increased distribution of cells, that is responsible for gliosis development. It is still unclear if the cell distribution is caused by the retinal breaks themselves or by the surgical treatment. [23] A study of Campo et al. from 1999 showed a postoperative gliosis in 16% of vitrectomized patients, 6% received peeling surgery subsequently. [28] Similar results were shown in this study with a peeling surgery rate of 7.3%.

The majority of patients (92.3%) described clear detachment symptoms, nevertheless they consulted a doctor on average after two weeks. In a work of Heussen et al, over one week passed till patients came to see a doctor. [29] According to Hassan et al., patients with retinal detachment treated surgically within 10 days after beginning of symptoms, have the highest visual increase postoperatively. [30] RD surgery performed after 6 weeks of symptom beginning leads to a significantly worse visual prognosis [31, 32].

RD of the second eye was seen in 7.1% of the cases within 6 years. Literature shows a large range of second eye RD appearance with 7-33%. [6] A careful examination of the fellow eye and education of the patient is therefore necessary. Cirulo et al. examined fellow eyes of 534 patients with unilateral RRD. Degenerated areas were seen in 90% of the patients, 20% already had one or more retinal breaks. [33]

A limitation of the study is the retrospective design and the large difference in the number of subjects in the PPV and SB group and the large variety of the treatment procedures. However, this study shows the range of RD surgeries performed during 2006 to 2008 in a regular clinical setting of a tertiary eye clinic with a long follow-up period.

## 8. Conclusion

A follow-up time of 6 months to 1 year seems appropriate to analyze redetachment rates; as in our 5-year-follow-up study, redetachments mostly occurred within the first 4 postoperative months. The hypothesis that eyes treated with SB might show a long-term risk of redetachment because of the remaining pathologic vitreous body could not be confirmed, but this statement has limited power because of the small number of patients in SB group. The study shows an increased ERM development after intraoperative use of endolaser cerclage in patients treated with vitrectomy. Interestingly 15% of the SB patients received a peeling surgery in the follow-up period. A routinely use of an endolaser cerclage might not be advisable. An encircling band does not seem to be of advantage in patients RRD up to medium complexity treated with PPV. Surgical treatment of RRD remains a controversial and highly individual decision influenced by the initial anatomical situation, patient characteristics, and the experience and ability of the surgeons. PPV seems to be appropriate in most patients with RRD. However, due to the high progression rate of cataracts, SB surgery is still recommended in younger patients and retinal breaks that may be clearly identified. In addition, a significant list of intraocular complications may be produced using PPV.

## Figures and Tables

**Table 1:** Intra- and/or postoperative complications of SB versus PPV.

*SB* ^*1*^ * (5 of 19, 26.3%)*	*n*	*PPV* ^*2*^ * (36 of 136, 26.5%)*	*n*

		*Intraoperative*	
		IOL^3^-luxation	2
		Haemorrhage of vitreous body, cortical remnants	2
		No success of vitreous body detachment	1
		Iatrogenic Foramina	5
		Iriscapture	1
		Haemorrhage in anterior chamber	1
		Macular hole	2
		PFCL^4^ on IOL^3^	1
		PFCL^4^ in anterior chamber	1
*Postoperative*		*Postoperative*	
Conjunctival dehiscence	1	Conjunctival dehiscence	2
Diplopia	1	Residual PFCL^4^	1
Buckle migration	2	Metamorphopsia	7
Metamorphopsia	1	Anterior chamber fibrinous reaction	1
		Macular scar	1
		Macular edema	6

1: SB= scleral buckle

2: PPV= Pars Plana Vitrectomy

3: IOL=intraocular lens

4: PFCL: Perfluorocarbon.

## Data Availability

The data used to support the findings of this study are available from the corresponding author upon request.
